# Comparison and Analysis of Zinc and Cobalt-Based Systems as Catalytic Entities for the Hydration of Carbon Dioxide

**DOI:** 10.1371/journal.pone.0066187

**Published:** 2013-06-20

**Authors:** Edmond Y. Lau, Sergio E. Wong, Sarah E. Baker, Jane P. Bearinger, Lucas Koziol, Carlos A. Valdez, Joseph H. Satcher, Roger D. Aines, Felice C. Lightstone

**Affiliations:** Physical and Life Sciences Directorate, Lawrence Livermore National Laboratory, Livermore, California, United States of America; University of Calgary, Canada

## Abstract

In nature, the zinc metalloenzyme carbonic anhydrase II (CAII) efficiently catalyzes the conversion of carbon dioxide (CO_2_) to bicarbonate under physiological conditions. Many research efforts have been directed towards the development of small molecule mimetics that can facilitate this process and thus have a beneficial environmental impact, but these efforts have met very limited success. Herein, we undertook quantum mechanical calculations of four mimetics, 1,5,9-triazacyclododedacane, 1,4,7,10-tetraazacyclododedacane, tris(4,5-dimethyl-2-imidazolyl)phosphine, and tris(2-benzimidazolylmethyl)amine, in their complexed form either with the Zn^2+^ or the Co^2+^ ion and studied their reaction coordinate for CO_2_ hydration. These calculations demonstrated that the ability of the complex to maintain a tetrahedral geometry and bind bicarbonate in a unidentate manner were vital for the hydration reaction to proceed favorably. Furthermore, these calculations show that the catalytic activity of the examined zinc complexes was insensitive to coordination states for zinc, while coordination states above four were found to have an unfavorable effect on product release for the cobalt counterparts.

## Introduction

In recent years a growing awareness of carbon dioxide atmospheric levels sparked interest in developing rapid methods for the capture and sequestration of the gas from industrial gas streams [1]. Most industrial separation processes for CO_2_ involve a liquid in which the dissolved gas ionizes under highly basic conditions, leading to its full dissolution and concomitant adsorption into the medium [2]. The rate-limiting step in such processes is well known to be the formation of carbonic acid. The slow kinetics nature of this reaction also hinders the uptake of CO_2_ in the ocean, and it is the underlying cause of the significant mass transfer limitation at the water’s surface [3]. This mass transfer limitation also applies to industrial gas separations [4], [5], [6] and results in overall decreases by a factor of 1000-fold over that which could be obtained, if the hydration of the CO_2_ was not the rate-limiting step. Accelerating such processes through the use of catalysts or enzymes would permit smaller and less expensive separation processes to remove CO_2_ from industrial gas emissions [7] and could conceivably be fast enough to permit removal of CO_2_ from the atmosphere in processes of the type envisioned by Elliot *et al* [8] and Keith *et al* [9].

In biological systems the reversible hydration of CO_2_ to bicarbonate is carried out with formidable efficiency by the zinc metalloenzyme, carbonic anhydrase (CA) [10]. In humans, carbonic anhydrase II (CAII, EC 4.2.1.1) is the most efficient isoform exhibiting activity that approaches diffusion limited kinetics. The reaction is catalyzed by a zinc-hydroxide containing center that is formed upon deprotonation of a water molecule coordinated to the active site’s zinc (Zn-OH_2_, p*K*_a_ ∼7) [11]. The reaction mechanism, which follows ping-pong kinetics, occurs via two independent steps [10], [12]. In step one, the zinc-hydroxide in the active site of CA nucleophilically attacks CO_2_ to form a Zn^2+^ bound bicarbonate intermediate whose reaction with water results in the expulsion of bicarbonate.
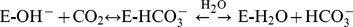
(1)


In the second step, the zinc bound water is deprotonated by a nearby histidine (His64 in human CAII) regenerating the catalytic species while the proton is shuttled into the bulk solvent.
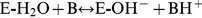
(2)


Deprotonation of the water is the rate-limiting step in carbonic anhydrase [12]. The extremely high hydration turnover of CO_2_ by human CAII is ∼10^6^ sec^−1^ at pH 9 and 25°C [11], [13]. The reverse reaction, dehydration of bicarbonate occurs when the solution pH is below 7.

The X-ray crystal structures of different CAs have been solved and studied in great detail [10]. Crystallographic studies of human CAII show that the enzyme is a monomeric protein consisting of 260 residues. The funnel-shaped appearance of the active site ends with the zinc metal located in its very interior and tetrahedrally coordinated by three histidines (His94, His96, and His119) and a water/hydroxide molecule [14], [15]. The active site can be divided into a hydrophobic half (Val121, Val143, Leu198, and Trp209) necessary for CO_2_ binding and a hydrophilic half (His64 and Thr199), possessing residues and water molecules intimately involved in an intricate hydrogen bonding network for efficient proton shuttling during the last step of the catalysis. Other divalent metals (Cu^2+^, Hg^2+^, Fe^2+^, Cd^2+^, Ni^2+^, Co^2+^ and Mn^2+^) [16] can bind to CAII, but only Co^2+^ has wild-type catalytic efficiency (*k*_cat_/*K*_m_  = 8.7×10^7^ M^−1^s^−1^ for Zn^2+^ vs 8.8×10^7^ M^−1^s^−1^ for Co^2+^), although the individual *k*_cat_ and *K*_m_ values for CAII differ when binding the two metal ions [17]. Due to the lack of spectroscopic signatures by the Zn^2+^ ion, its divalent counterpart Co^2+^ has played an important role in studying CA, not only because it also utilizes metal-hydroxide catalysis and retains near wild-type activity but because it also acts as a spectroscopically active tag [18].

**Figure 1:**
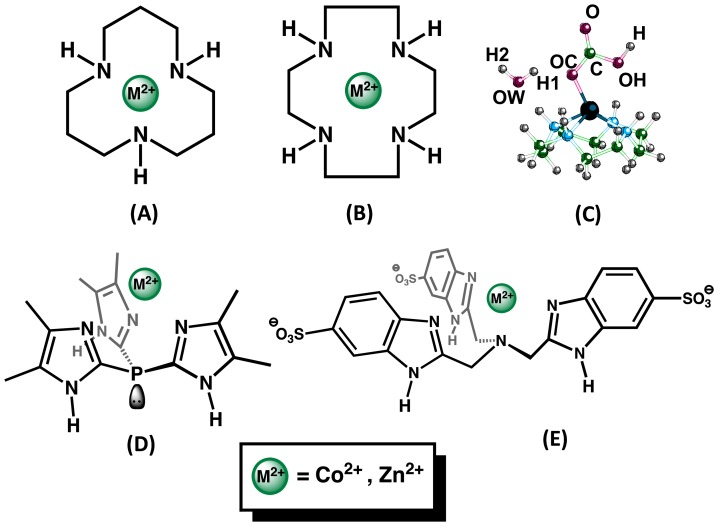
Molecular structures of 1,5,9-triazacyclododedacane (A) and 1,4,7,10-tetraazacyclododedacane (B) which are denoted N3 and N4 in the text, respectively. Panel (C) shows how the atoms are denoted in the text. The structures of tris(4,5-dimethyl-2-imidazolyl)phosphine (D) and tris(2-benzimidazlylmethyl)amine (E) are denoted Ph and Ben in the text, respectively.

Despite the merits of CAII, current research into the use of carbonic anhydrase for industrial CO_2_ capture has faced significant challenges mainly due to the challenging task of producing a viable enzyme for the rigorous demands encountered in industrial processes. Trachtenberg et al [7], [19] have reported the use of a membrane-countercurrent system originally designed for spacecraft use, and Bhattacharya et al [20] developed a spray system containing carbonic anhydrase. Azari and Nemat-Gorgani [21] examined means of using the reversible unfolding of the enzyme, caused by heat, to attach it to more sturdy substrates for industrial use. Lastly, Yan et al [22] incorporated single carbonic anhydrase molecules in a spherical nanogel, resulting in improved temperature stability of the enzyme with only moderate loss of activity. Another route of exploration and one that has been undertaken by several groups is to synthesize small molecules capable of mimicking the enzyme’s catalytic property. Creating such mimetics requires incorporating key structural features from the enzyme scaffold and avoiding possible degradation mechanisms of the catalytic center. Fortunately, CA mimetics were developed to study the enzyme’s reaction mechanism, and several examples of small molecule CA mimetics exist [23]. In the small molecule mimetics developed to date, the most prominent features of the enzyme’s catalytic site, namely the nitrogen atoms belonging to the histidine side chains, have been used as guiding factors in their design. These nitrogen atoms may be part of an imidazole group [24], such as tris(4,5-di-n-propyl-2-imidazolyl)phosphine or nitrilotris(2-benimidazolylmethyl-6-sulfonate), or simply secondary amines, as in to case of 1,5,9-triazacyclododecane [25] or 1,4,7,10-tetraazacyclododecane [26] which chelate the a metal ion to form the catalytic species (Figure 1). These four small molecule mimetics when chelated with Zn^2+^ have been reported to catalyze the hydration of CO_2_, although with a more modest catalytic activity compared to the enzyme.

In this *ab initio* study, we have examined carbon dioxide hydration as catalyzed by 1,4,7,10-tetraazacyclododedacane (**N4**), 1,5,9-triazacyclododedacane (**N3**), tris(4,5-dimethyl-2-imidazolyl)phosphine (**Ph**), and tris(2-benzimidazolylmethyl)amine (**Ben**), chelating both Zn^2+^ and Co^2+^, to investigate the reaction mechanism of these two metals and determine the cause for the difference in activity seen in human CAII.

## Methods

### Quantum Mechanical Calculations

The hydration reaction of CO_2_ catalyzed by **N3**, **N4, Ph,** and **Ben,** chelating Zn^2+^ and Co^2+^, was investigated using quantum mechanical calculations. All calculations were carried out using the programs Gaussian03 [27] and Gaussian 09 [28]. Geometry optimizations were performed at the B3LYP/6-311+G(d) level of theory [29], [30]. The catalytically active form of cobalt in carbonic anhydrase is experimentally known to be a high-spin quartet (S = 3/2) [31]. Ground state calculations of these mimetics containing low-spin (S = 1/2) Co^2+^ were consistently higher in energy than the high-spin system. Thus, calculations on the cobalt-containing mimetics were carried out with a fixed quartet multiplicity. The stability of the wavefunction was determined by using the STABLE option within Gaussian. The counterpoise method of Boys and Bernardi was used to account for basis set superposition error (BSSE) [32]. To test the suitability of the B3LYP functional for these calculations, full optimizations of **N4**-metal reaction were performed, using a recent functional (MPWLYP1M/6-311+G(d)) that has been successfully used for several organometallics (Figure S1) [33]. Harmonic frequency calculations were performed on all the structures to characterize the stationary points. Transition states were characterized by a single imaginary frequency, and their values are provided in Table S1. The calculated zero-point energies (ZPE) were not scaled. To investigate the effects of solvation on the hydration reaction, single point calculations using the gas-phase geometries were carried out, using a conductor-like polarizable continuum model (CPCM) [34] to approximate solvent effects (water, ε  = 78.4). It has been previously shown that the solvation free energies from single point PCM calculations, using gas-phase geometries from density functional calculations, are in reasonable agreement with values obtained from full optimizations [35], [36]. All solvation calculations used the simple united atom topological model (UA0) [37], using UFF radii [38]. The gas phase zero point energies were included in the solvation calculations. Natural population analysis was performed on the optimized structures to assess the charge distributions on these complexes [39].

### Synthesis

#### Tris(6-sulfobenzimidazolylmethyl)amine (sulfonated-Ben)

The ligand was synthesized following a previously published protocol for the synthesis of tris-benzimidazole-based compounds [40]. Thus, 4-sulfo-1,2-diaminobenzene [41] (4.0 g, 21.2 mmol) was transferred into a 250 mL round bottom flask equipped with a large stir bar. The solid was made into a suspension with the addition of ethylene glycol (120 mL). To the suspension, nitrilotriacetic acid (1.13 g, 5.89 mmol) was added in one portion, the flask equipped with a condenser (set with water at 10°C) and the resulting mixture was heated to 210°C, using a sand bath overnight. After 18 hours of heating, the flask was removed from the sand bath, and the black-colored reaction mixture was allowed to cool down to ambient temperature. The mixture was subsequently poured into a 1000 mL Erlenmeyer flask containing ice water (300 mL) in small portions with constant swirling. The grey precipitate was collected using vacuum filtration and washed copiously with cold, deionized water (5×50 mL) and dried under vacuum to afford the title compound (2.90 g, 74%). The sodium salt of the ligand was obtained by reacting the ligand (1.0 g, 1.5 mmol) with NaOH (180 mg, 4.5 mmol, 3.05 equiv. to ligand) in deionized water (10 mL). The water was evaporated under reduced pressure to yield a light grey solid (1.03 g, 97%). ^1^H NMR (600 MHz, D_2_O) δ 7.94 (s, 3H), 7.53 (d, *J*  = 8.5, 3H), 7.35 (d, *J*  = 8.5, 3H), 4.07 (s, 6H); ^13^C NMR (150 MHz, D_2_O) δ 155.4, 139.6, 137.9, 136.5, 119.7, 114.6, 112.8, 53.2; Anal. (C_24_H_18_N_7_Na_3_O_9_S_3_•H_2_O) C, 39.40; H, 2.76; N, 13.40; Found: C, 39.32; H, 3.08; N, 13.49. The characterization of the zinc complex and the protocols for the kinetic analysis for sulfonated-**Ben** can be found in Text S1.

## Results and Discussion

Our *ab initio* calculations investigated the hydration of CO_2_ catalyzed by the Zn^2+^ containing catalysts (Figure 1). The hydration of CO_2_ by carbonic anhydrase and mimetics is believed to follow the same reaction pathway. Thus, the catalytic cycle begins with nucleophilic attack on the CO_2_ by the zinc-hydroxide species to form Zn^2+^-bicarbonate intermediate followed by displacement of the bicarbonate from Zn^2+^ by water; the water then loses a proton to regenerate the catalysis. Cobalt substituted carbonic anhydrase also utilizes the above metal-hydroxide reaction mechanism for CO_2_ hydration, but it has ∼50% of the catalytic activity exhibited by the wild-type enzyme [17]. To gain insight in the possible fine differences between the two metals, chelators containing Co^2+^ were also studied. The cobalt complexes are assumed to be in alkaline conditions which favor tetrahedral geometries and share similar characteristics to the zinc complexes [42], [43]. There have been several *ab initio* studies on the hydration of CO_2_ by CAII [44], [45], [46], [47], [48], [49] but a thorough comparative study between Zn^2+^ and other metals ions within CA have not been as widely studied [50], [51], [52]. Additionally, tris(4,5-di-n-propyl-2-imidazolyl)phosphine catalyzes the hydration of CO_2_, but the non-catalytic tris(4,5-dimethyl-2-imidazolyl)phosphine (**Ph**) was chosen for computational tractability and can provide insights into the reaction mechanism.

### Nucleophilic Attack of CO_2_

**Figure 2:**
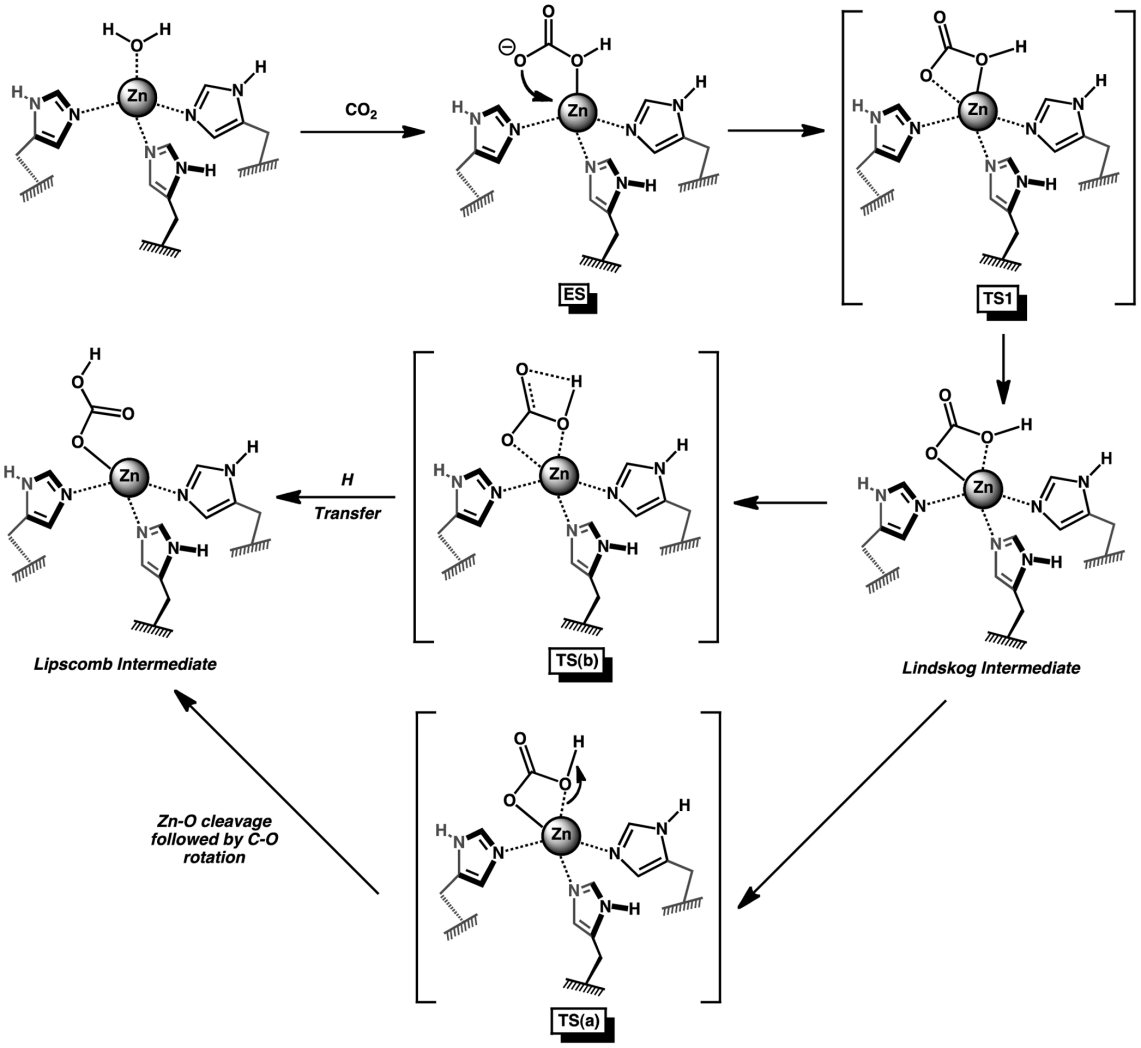
Schematic of the nucleophilic attack of CO_2_ by the mimetics and the resulting geometries of the Lindskog and Lipscomb intermediates.

**Figure 3:**
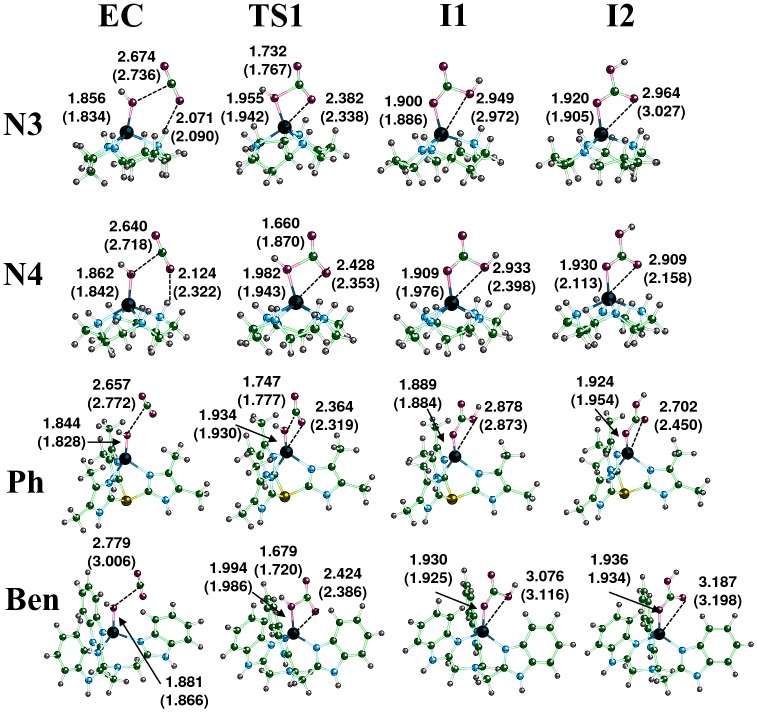
Calculated structures for the zinc complexes of N3, N4, Ph, and Ben complexes during the nucleophilic attack portion of the hydration reaction of CO_2_ when forming the encounter complex (EC), first transition state (TS1), Lindskog intermediate (I1), and Lipscomb intermediate (I2). Distances are listed in angstroms and values in the parenthesis are the corresponding distances for the cobalt complexes.

**Figure 4:**
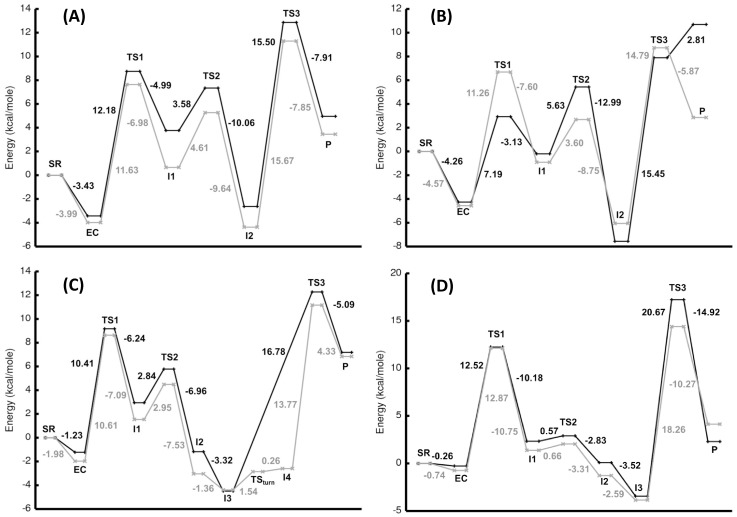
Relative energy of the calculated stationary points for N3 in Panel (A), N4 in Panel (B), Ph in Panel (C), and Ben in Panel (D) along the reaction coordinate relative to the separated reactants (SR). The energies for the zinc complexes are represented by the gray line and the cobalt complexes by the black line.

The first step of the catalyzed hydrolysis of CO_2_ in the gas-phase is formation of an encounter complex (EC) between the separated reactants (Figure 2). The EC is formed when CO_2_ interacts weakly with one of the amine hydrogens in the ring structure of the macrocycles **N3** and **N4** (Figure 3). Due to the lack of N-H moieties around the catalytic OH^-^ group in the **Ph** and **Ben** ligands, only van der Waals complexes were formed with CO_2_. The stabilization energy is approximately −1 to −4 kcal/mole for each of the Zn^2+^ and Co^2+^encounter complexes relative to the separated reactants (Figures 4). The **N3** and **N4** ECs were found to have greater stabilization energies than the **Ph** and **Ben** ECs. The amine hydrogen to CO_2_ oxygen distances were measured to be 2.071 and 2.124 Å for **N3**-Zn and **N4**-Zn, respectively, while the Co^2+^ complexes had similar distances of 2.090 Å (**N3**-Co) and 2.322 Å (**N4**-Co). Additionally, the angle formed by the CO_2_ oxygen with the hydrogen and nitrogen of the amine (O••• H-N) of **N3** and **N4** shows that the CO_2_ is likely not forming a strong hydrogen bond. Both **N3** M^2+^-complexes have angles close to 180°, whereas the **N4** complexes possess angles of 137° for Co^2+^ and 158° for Zn^2+^. The distances between the CO_2_ carbon and the M^2+^-hydroxide oxygen were 2.674 (**N3**) and 2.640 Å (**N4**) for the Zn^2+^ EC structures, while the Co^2+^ complexes had slightly longer distances. The value obtained for **N3**-Zn is similar to that obtained by Brauer et al. at the HF/6-311+G(d) level of theory, while our **N4**-Zn value is almost 0.1 Å shorter than Brauer’s value [53]. Calculations using the B3LYP or MPWLYP1M functional provide similar results for both M^2+^-complexes. Only minor differences in the energies and geometries were found for the **N4** reaction with either Zn^2+^ or Co^2+^ between these fully optimized calculations (Figure S1).

The calculated distances for the EC structures are in reasonable agreement with a recently solved crystal structure of human carbonic anhydrase (HCAII) with CO_2_ [54]. In this crystal structure (PDB ID 3D92), the CO_2_ carbon to Zn^2+^-hydroxide oxygen distance is 2.791 Å. The CO_2_ is bound in a hydrophobic pocket within HCAII, and one of its oxygens interacts with the amide backbone nitrogen of Thr199 (3.493 Å). Interestingly, the same study also showed that a metal ion is not even necessary for CO_2_ to bind in the correct location in the HCAII active site. Although **Ph**-metal and **Ben**-metal complexes lack an N-H group to stabilize the CO_2_ around the ligands, the distance from the M^2+^-hydroxide oxygen to the CO_2_ carbon was comparable to the **N3** and **N4** ligands, even though their stabilization energy is smaller (Figure 3). Natural population analysis (NPA) of the complexes show that there is little charge difference between Zn^2+^ in **N3** or **N4** (Table S2), a finding that is consistent with the work of Brauer *et al* [53]. Two additional pieces of information obtained from NPA are: (1) there exists some charge polarization occurring in the CO_2_ molecule from its interaction with the amine and (2) the Zn^2+^-hydroxide is more nucleophilic in nature than its Co^2+^ counterpart.

The first transition state (TS1) in the hydration reaction is formed when the distance between the M^2+^-hydroxide oxygen to the carbon of CO_2_ falls below 2 Å. In the Co^2+^ complexes, the distances for the **N3**-Co, **N4**-Co, **Ph**-Co, and **Ben**-Co transition states were 1.767, 1.870, 1.777, and 1.720 Å, respectively (Figure 3). The **N3**-Zn complex has a similar transition state distance to **N3**-Co of 1.732 Å, but the **N4**-Zn structure has a much shorter distance of 1.660 Å relative to **N4**-Co. The reaction barriers are similar for **N3**-Zn, **N4**-Zn, and **N3**-Co (∼12 kcal/mole), while the reaction barrier for **N4**-Co is significantly lower at 7.2 kcal/mole (Figure 4B). The difference in energy is due to an earlier transition state for **N4**-Co than found in the other complexes. Although the CO_2_ to M^2+^-hydroxide distance in **N4**-Co is longer than in **N4**-Zn, the oxygen in CO_2_ shows greater coordination to Co^2+^ (2.353 Å) relative to Zn^2+^ (2.428 Å). The TS1 geometries for both **Ph**-metal structures are similar. The distance between the hydroxyl oxygen and carbon of CO_2_ is 1.747 for **Ph**-Zn. Both **Ben**-metal structures had late transition states that lead to high activation barriers for the final formation of bicarbonate (∼13 kcal/mole). There is minimal change in the charge on either metal in going from EC to TS1 except for **Ph**-Co. When TS1 is formed, the charge on the hydroxyl oxygen drops to almost the same values (ranging from −1.02 to −1.09 |eu|) for all eight complexes even though the Zn^2+^ complexes were found to possess higher charges in the EC structures (Table S2).

After passing the first transition state, a bicarbonate complex directly chelated to the metal is formed. There has been great deal of debate about the actual conformation of the bicarbonate around the metal center in carbonic anhydrase. From these calculations and others, a Lindskog intermediate (OH of bicarbonate is oriented towards the metal, Figure 2) will clearly be formed first in this reaction (denoted I1) [51], [53], [55]. The geometry of both **N3** complexes is very similar with one oxygen directly coordinated to the metal center (1.886 Å and 1.900 Å for Co^2+^ and Zn^2+^, respectively), and the bicarbonate hydroxyl group weakly interacting with the metal ion (2.972 Å and 2.949 Å for Co^2+^ and Zn^2+^, respectively). The geometry around the metal is tetrahedral. Similar asymmetrical bicarbonate coordination geometries for I1 were obtained for the **Ph** and **Ben** complexes for both metal ions (Figure 3). The **N4**-Zn structure resembles the **N3**-metal structures with a single oxygen coordinated to Zn^2+^, and the hydroxyl group asymmetrically interacting with the zinc (1.909 and 2.933 Å). The I1 **N4**-Co geometry differs from the other complexes. The oxygens coordinating Co^2+^ are much more symmetrical. The metal to coordinating oxygen distance is 1.976 Å, and the hydroxyl oxygen is 2.398 Å away. Although not perfectly octahedral, this structure shows cobalt’s ability/preference to coordinate six ligands.

Rotation about the oxygen bond coordinated to the metal center in the Lindskog intermediate (I1) leads through a shallow transition state (TS2) to the lower energy Lipscomb intermediate (I2), which has both carboxylate oxygens of bicarbonate directed towards the metal [56]. This second transition state occurs when the dihedral angle (OC – C, see Figure 1C) has rotated approximately 90°. For both metal ion complexes of **N3, Ph,** and **Ben** structures, TS2 has almost identical geometries. Interestingly, the TS2 structures for **N4** differ significantly (Figure S2). **N4**-Zn has a transition state that resembles the **N3** structures, but the **N4**-Co TS2 structure still shows a preference for octahedral binding even though one site is unoccupied. It should be pointed out that proton transfer from the hydroxyl oxygen (OH) of bicarbonate to the non-coordinated oxygen (O) is also a viable mechanism for conversion of I1 to I2 but requires additional water molecules for this to have an activation barrier as low as bond rotation [57]. In either case, this portion of the reaction is not expected to be rate-limiting.

The Lipscomb intermediates for both **N3** complexes are similar; a single oxygen is coordinated to the metal, and the other carboxylate oxygen is weakly coordinated to the metal (1.920 and 2.964 Å for Zn^2+^ and 1.905 and 3.027 Å for Co^2+^). For the **N4** complexes, the carboxylates of bicarbonate are also bound differently in the Lipscomb intermediate depending on the metal. For **N4**-Zn, the oxygen to zinc distances are 1.930 and 2.909 Å which is similar to the values for the unidentate **N3**-Zn complex. For **N4**-Co, the oxygen cobalt distances are almost identical at 2.113 and 2.158 Å, again reflecting cobalt’s preference for an octahedral geometry in this macrocycle. The bicarbonate geometries for the **Ph** compounds were unidentate for Zn^2+^ but bidentate for Co^2+^. These calculated results are in good agreement with crystal structure data of Zn^2+^ and Co^2+^ bound by a tris(pyrazoyl)hydroborato ligand and coordinating nitrate or carbonate [58], [59]. In the Zn^2+^ compounds, only one nitrate or carbonate oxygen binds to the metal at a distance of 1.98 Å, and the second oxygen is greater than 2.6 Å from the metal. For the Co^2+^ compounds, the two oxygens bind more symmetrically around the metal at 2.001 and 2.339 Å for nitrate and 2.055 and 2.271 Å for carbonate in the crystal structures. The bicarbonate I2 geometries for the **Ben** compounds were almost identical. Both metals bind the bicarbonate in a unidentate geometry with oxygen distances of 1.936 and 3.187 Å for Zn^2+^ and 1.934 and 3.198 Å for Co^2+^.

The calculated results for nucleophilic attack of CO_2_ are in qualitative agreement with model studies. The x-ray crystal geometries of tris(pyrazolyl)hydroborato zinc hydroxide and cobalt hydroxide complexes are similar to those obtained for the **N3** complexes [60]. These structures all have tetrahedral geometries around the metal center and readily react with CO_2_ to form bicarbonate. Unfortunately, the tris(pyrazolyl)hydroborato complexes are not soluble in water therefore release of the metal bound bicarbonate is not possible with these catalysts.

### Product Release

**Figure 5:**
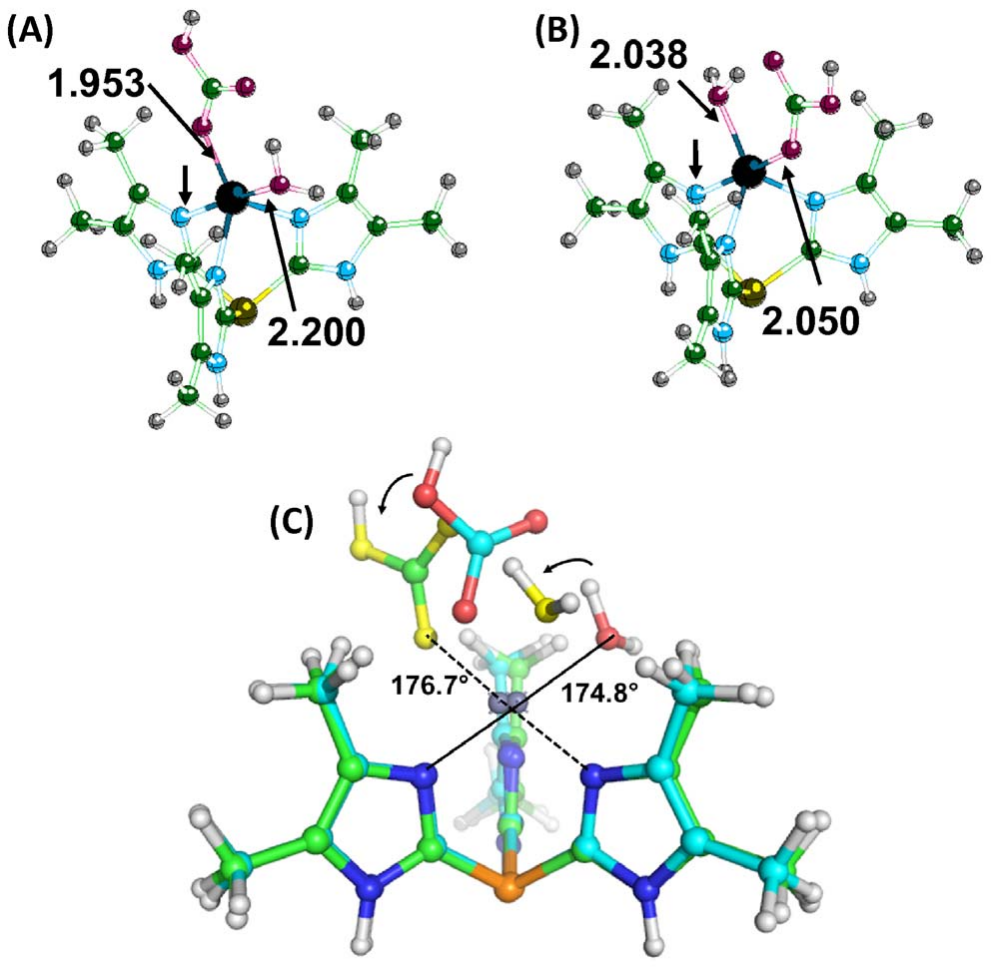
Optimized structures of the Ph-Zn-bicarbonate-water complexes. Panel (A) is the lowest energy structure and has the water in the axial position. The angle formed by the imidazole nitrogen (arrow)-Zn-oxygen (water) is almost linear. Panel (B) has the bicarbonate in the axial position. Panel (C) shows an overlay of the two structures and how interconversion between the two geometries can occur.

To study the release of bicarbonate from the metal center, a single water molecule was added to the I1 (Lindskog) and I2 (Lipscomb) structures since it was not obvious which geometry would have the lower activation barrier for product release. Once a water molecule was added to each intermediate, the structures were reoptimized. In all cases, the I2 structure with water was the lower energy structure. The water molecule was stabilized by formation of a hydrogen bond between the oxygen of water (OW) and the hydrogen from the amine group in the ring structure of both **N3** and **N4** and interaction of a hydrogen from water (H1) with the oxygen of bicarbonate (OC) coordinating the metal (Figure S3). For the **Ph** and **Ben** structures, the water hydrogen bonds with the bicarbonate but likely does not interact strongly with the rest of the complex since there are no other polar groups in the vicinity. Interestingly, the structure obtained for the **Ph**-Zn is very similar to the x-ray structures of 2VVB [61] and 1XEG [62], where a unidentate bicarbonate or acetate is interacting with one water molecule. The original intermediate structures were not significantly affected by the inclusion of the water molecule. The energy difference of I1 relative to I2 did not change significantly by adding the water molecule. Unlike the macrocycles, lower energy structures than the encounter complex were found when the water molecule directly coordinates to the metal ion for both **Ph** and **Ben** complexes. When the water coordinates to the metal ion in the **Ph** complex, a trigonal bipyramidal metal center is formed. Two conformers are possible in the case of zinc, the lowest energy structure has the water in the axial position and the bicarbonate (in the equatorial position) hydrogen bonds to the coordinated water (Figure 5A). Although it is possible to obtain a minimum energy structure with bicarbonate in the axial position and water equatorial (Figure 5B), the energy barrier (TS_turn_) separating these two structures disappears when the ZPE correction is included. A turnstile pseudorotation occurs to transform one structure to the other, but the amount of rearrangement to have the bicarbonate go from axial to equatorial is small because of the three-fold symmetry of the phosphine complex (Figure S4). Both ligands only need to rotate by ∼60° to interconvert between conformations. Similar coordination of water and bicarbonate around the zinc has also been observed in carbonic anhydrase II binding acetate (Figure S5).

In the wild-type (WT) protein (PDB, 1CAY), the coordination around the zinc was a distorted trigonal bipyrimid with a water as an equatorial ligand and the acetate as an axial ligand [63]. Comparison of the WT enzyme with the T199A mutant (PDB, 1CAM) shows that the hydroxyl group of T199 is important in the positioning of the water molecule coordinated to the zinc (Figure S6) [64]. The positioning of the water and the bicarbonate around the zinc by Thr199 likely creates a situation in which the geometry is not optimal and makes release of bicarbonate more favorable. The Zn-carboxylate oxygen distances for the WT and T199A proteins are 2.42 and 2.27 Å, respectively. In the 1CAM crystal structure, the angle formed by the His94 NE2– Zn – O of water is 136.6°. The analogous angle in 1CAY is 110.0°. Experimentally, the T199A mutant is ∼100 times slower at turning over CO_2_ than the WT enzyme, and the binding of inhibitors such as thiocyanate and bicarbonate is enhanced by 20-fold [65]. In the E106Q mutant protein (PDB, 1CAZ) [63], the zinc coordination is trigonal bipyramidal, but the water and acetate coordination is now reversed with water as the axial ligand and acetate as the equatorial ligand (Figure S5). These calculations show bicarbonate is more strongly coordinated to the Zn^2+^ in the equatorial position (Zn-O bond is 1.953 Å) relative to the axial position (Zn-O bond is 2.050 Å) in **Ph**-Zn and the lowest energy conformation for the trigonal bipyramidial geometry. The carboxylate sidechain of E106 is important for positioning the water around the zinc to avoid this conformation since the E106D mutant shows little change in activity from the wild-type enzyme. The amide sidechain of E106Q rotates away from T199 and functions as a hydrogen donor with T199 instead of an acceptor. This changes the hydrogen bonding network within the active site, resulting in a 1000-fold decrease in the maximal rate for the E106Q mutant [65].

**Figure 6:**
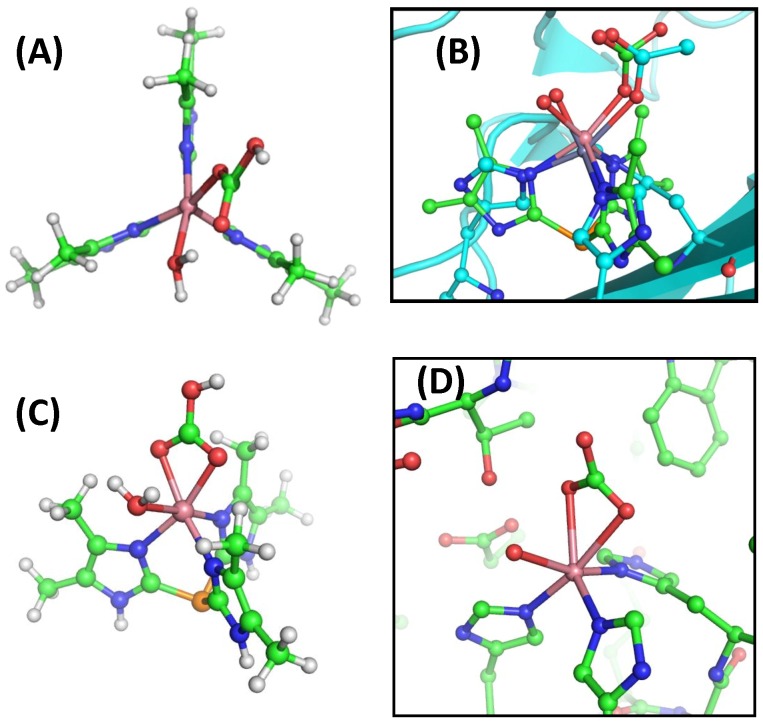
Optimized structures of Ph-Co-bicarbonate-water complexes. Panel (A) shows the low energy structure of **Ph**-Co interacting with water (I3). Panel (B) shows an overlay of the **Ph**-Co (I3, green) structure with the wild-type (zinc) carbonic anhydrase with acetate (1XEG, cyan). Panel (C) shows an octahedral geometry for bicarbonate, and water bound to cobalt. Panel (D) shows the arrangement of ligands (water and bicarbonate) around the metal ion in the X-ray crystal structure of cobalt carbonic anhydrase (1CAH).

The **Ph**-Co encounter complex is a distorted trigonal bipyramidal structure with water in the axial position and bicarbonate in the equatorial position. One oxygen of the bicarbonate is hydrogen bonding with the water molecule. This complex could also be described as having an octahedral geometry but missing the sixth ligand. Interestingly, a formal octahedral complex 3.6 kcal/mole higher in energy relative to the pentacoordinate structure was also obtained. This structure, which has two oxygens of the bicarbonate equatorially coordinated to Co^2+^ and water at the axial position, is almost identical to the Co^2+^ carbonic anhydrase binding bicarbonate (PDB 1CAH) [66] (Figure 6) and also the structure of cobalt tris[2-isopropylimidazol-4(5)-yl]phosphane coordinating to nitrate and water [67]. A similar octahedral structure was also obtained for the Zn^2+^ complex that was 5.44 kcal/mole higher in energy relative to the I3 structure. The trigonal bipyramidal coordination of the bicarbonate in the enzyme may not be favorable. An overlay of the I3 structure in the active site of 1CAH shows the bicarbonate would be in close contact with the side chain of L198.

**Figure 7:**
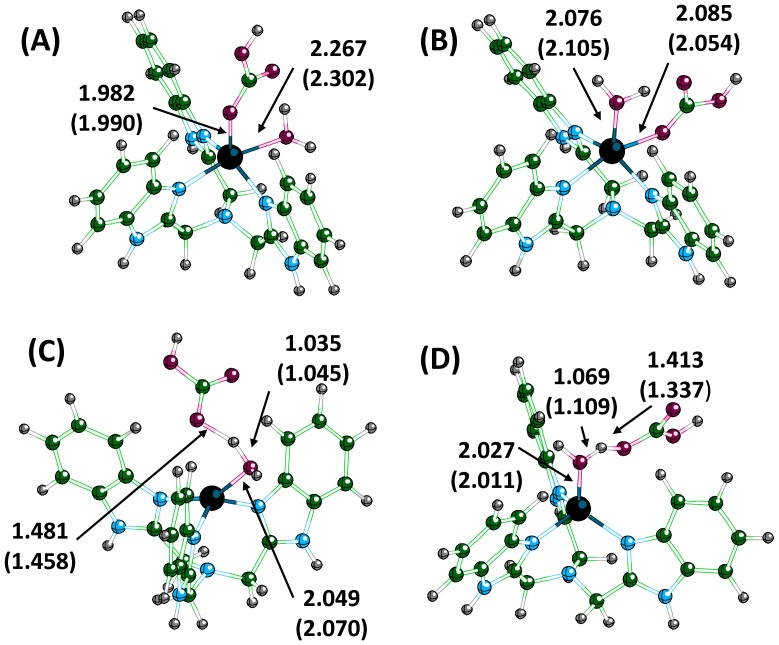
Optimized geometries for the bicarbonate and water bound to Ben-Zn with the bicarbonate in the equatorial position (A) and axial position (B). The equatorial structure is the most stable. Panel (C) and (D) show the corresponding transition states for (A) and (B), respectively. Values are in angstroms and values in parenthesis are for the corresponding cobalt structures.

Axial and equatorial arrangements for bicarbonate in both **Ben**-metal complexes were also found, with the equatorial geometry the more stable by ∼2 kcal/mole, but interconversion between these structures was not possible (Figure 7). When water coordinates to the metal, an octahedral complex forms for both **Ben-**metal complexes as the bicarbonate shift positions to take up an equatorial arrangement around the metal. The interconversion of conformation by the turnstile pseudorotation in this case would likely have a high barrier since this complex is not symmetric and would require the water molecule and bicarbonate to exchange positions (180° rotation). The octahedral geometry adopted by the **Ben**-Zn complex is reminiscent of tris(6-amino-2-pyridylmethyl)amine binding Zn^2+^ which catalyzes phosphodiester cleavage [68]. Indeed, complexes of Zn^2+^, Co^2+^, and Cu^2+^ coordinated to tris(2-benimidazylmethyl)amine have been shown to catalyze the hydrolysis of p-nitrophenyl acetate [69].

**Figure 8:**
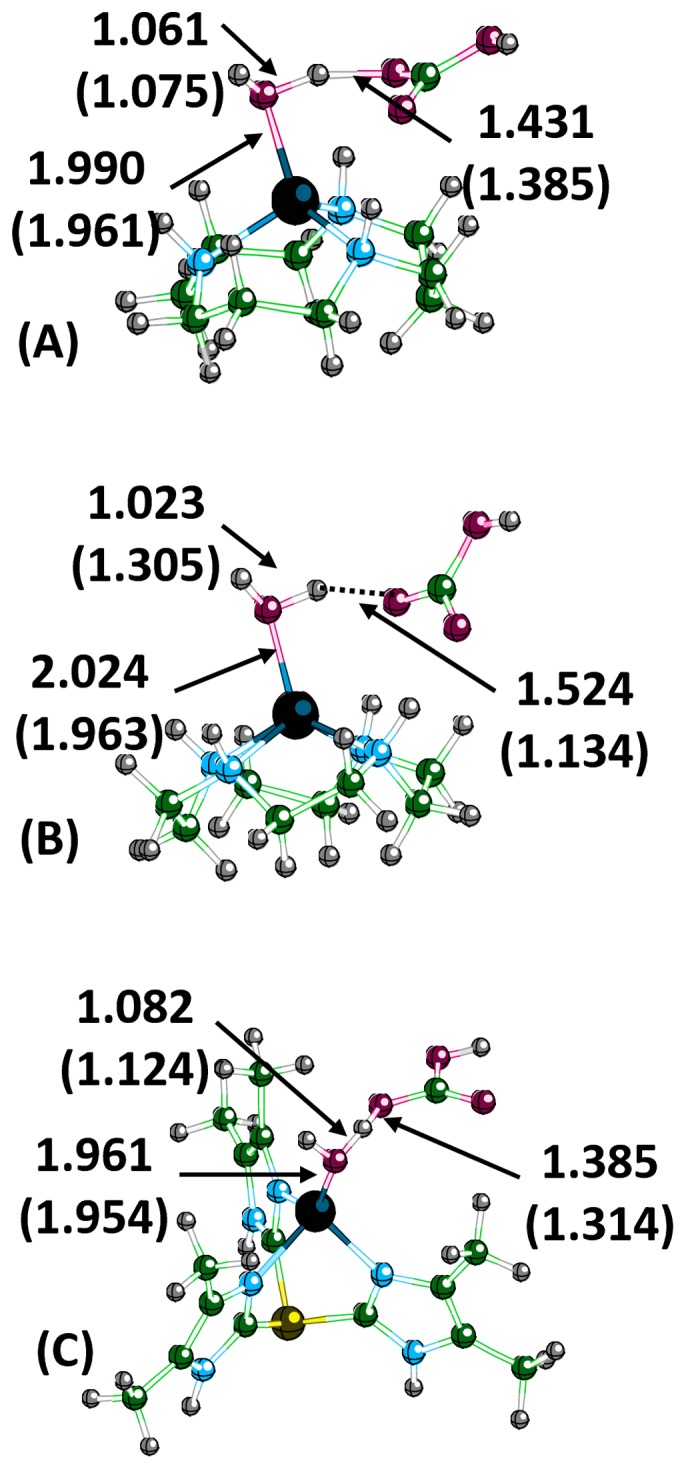
Calculated transition state structures (TS3) for displacement of bicarbonate by a water molecule for N3-Zn (A), N4-Zn (B), and Ph-Zn (C). Numerical values are in angstroms and values in parenthesis are the corresponding values for the cobalt structures.

For product release, an addition-substitution reaction occurs with a water molecule displacing the bicarbonate. At the transition state (TS3), the water molecule coordinates to the metal ion, causing the oxygen of the bicarbonate to weaken (Figure 7 and 8). In the zinc complexes, the oxygen of water is 1.900 Å (**N3**), 2.024 Å (**N4**), 1.961 Å (**Ph**), and 2.049 Å (**Ben**) from Zn^2+^ with the oxygen of the previously coordinated bicarbonate now at 2.938 Å (**N3**), 2.886 Å (**N4**), 2.676 Å (**Ph**), and 2.823 Å (**Ben**). The displaced oxygen of bicarbonate interacts with one of the hydrogens of the water and ultimately abstracts the proton from the water to form carbonic acid and to reform the metal-hydroxide catalyst. The transition state geometry for **N3**-Co is almost identical to **N3**-Zn, with the water bound slightly tighter (1.961 Å) and the bicarbonate more weakly bound to the metal (3.017 Å). The transition state for **N4**-Co differs from the other three structures. The water is bound tightly to the Co^2+^, and the oxygen of bicarbonate is still coordinated to the metal (2.509 Å). Additionally, the previously mentioned TS3 structures (both **N3** and **N4**-Zn) had the oxygen of bicarbonate interacting with one of the hydrogens on the water molecule. In the TS3 structure of **N4**-Co, the hydrogen/proton from the water has transferred to the bicarbonate to form carbonic acid. No change in the ring structure occurred for either **N4** complex at the transition state.

From these calculations, **Ben**-Zn should be a poorer catalyst at CO_2_ hydration that **N3** or **N4,** although the water soluble sulfonated version of **Ben**-Zn was reported to be highly active at low temperatures, and its activity was extrapolated to room temperature [70]. Interestingly, no direct kinetic measurements for the sulfonated-**Ben**-Zn were reported at room temperature. To better understand the catalytic properties of **Ben**-Zn, the sulfonated benzimidazole compound was synthesized and tested. The sulfonated-**Ben**-Zn complex did not show any catalytic properties at room temperature and had slight activity at 50°C (Figure S7), which was consistent with the calculated activation barrier for **Ben**-Zn.

For all complexes, the transition state from the I2 structure was lower in energy than the transition state from the I1 structure. Interestingly, the activation barrier from I1 or I2 to their respective transition states was almost identical in value. The activation barrier for bicarbonate release ranges from a low value of 14.8 kcal/mole for **N4**-Zn to a high value of 20.7 kcal/mole for **Ben**-Co. From these calculations, product release is the rate-limiting step for the hydration of CO_2_. These values differ from those obtained by Mauksch et al., using the model system [(NH_3_)_3_Zn(OH)]^+^/CO_2_ [71]. They find that nucleophilic attack is the rate-limiting step for CO_2_ hydration and only a small barrier for product release. This discrepancy is due to their assumption that one of the protons on the coordinated water molecule transfers to the bicarbonate while both are still coordinated to the zinc. This proton transfer seems unlikely in solution from p*K*_a_ measurement of the macrocycle triamine [2-(2-hydroxyphenyl)-1,5,9-triazacyclododecane coordinated with zinc [72]. This macrocycle is pentacoordinated with a trigonal bipyramidal geometry. The 2-hydroxyphenyl moiety has a p*K*_a_ of 6.8, and the coordinated water has a p*K*_a_ of 10.7. Having a charged oxygen coordinated to the zinc reduces the metal’s ability to acidify the water molecule since the p*K*_a_ of water bound to 1,5,9-triazacyclododecane-zinc is 7.5. The calculated activation barriers for the zinc complexes for **N4** (14.8 kcal/mole), **N3** (15.7 kcal/mole), **Ph** (15.6 kcal/mole), and **Ben** (18.3 kcal/mole) are in reasonable agreement with measured rate constants for CO_2_ hydration 2494 M^−1^ S^−1^ [^73^], 1083 M^−1^ S^−1^ [73], 898 M^−1^ S^−1^ [74], and not catalytic, respectively. The correlation between product release and experimental rate constants is consistent with our previous results, showing the bond dissociation energy between bicarbonate and Zn-azamacrocycles corresponds with the experimental results [73].

The calculated hydration reaction catalyzed by the tetrahedral coordinating **N3,** using either Zn^2+^ or Co^2+^, was very similar in both geometries and energies obtained. This is consistent with experimental results that show almost identical coordination geometries and wild-type activity for alpha-class carbonic anhydrases that have Zn^2+^ substituted with Co^2+^ [17], [75]. The calculated activation barrier for release of bicarbonate is high in these polyamine complexes yet HCAII experiments have shown this step in the reaction to be rapid and not rate-limiting [76]. In HCAII, both experiment and theory have shown that Thr199 has a destabilizing effect on bicarbonate binding to zinc [65], [77]. Hybrid QM/MM calculations by Merz and Banci show that the active site of HCAII promotes destabilization by pulling one of the carboxylate oxygens of bicarbonate away from the zinc by formation of a hydrogen bond with the hydroxyl group of Thr199 [78]. Using PM3 calculations, they found that having the zinc-bicarbonate active site geometry destabilizes the Lipscomb intermediate by 8.7 kcal/mole relative to the QM optimized structure. These results are also qualitatively in agreement with estimated free energies from kinetics data for carbonic anhydrase that show dissociation of bicarbonate limits the CO_2_ hydration catalyzed by HCAI and the Thr200His mutant of HCAII [79].

### Solvent Effects on CO_2_ Hydration

**Figure 9:**
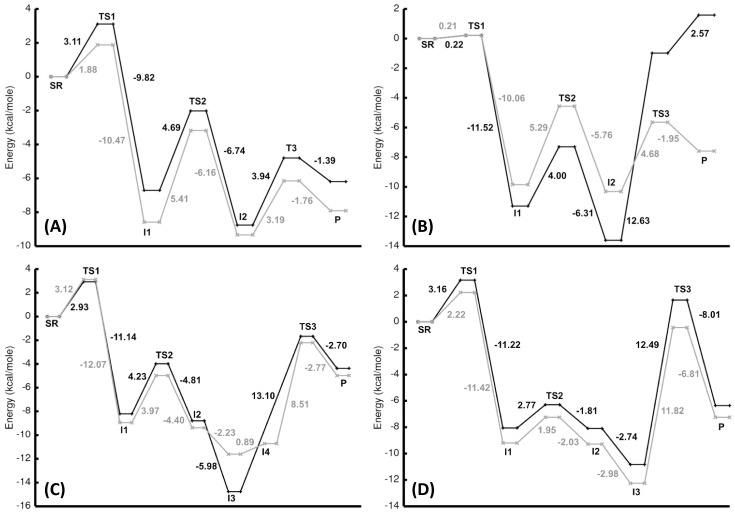
Relative energies to the separated reactants from single point solvation (CPCM) calculations using the gas-phase stationary points for the zinc (black) and cobalt (gray) complexes are shown for N3 (A), N4 (B), Ph (C), and Ben (D).

To estimate the effects of solvent on the CO_2_ hydration reaction, single-point conductor-like polarization continuum model (CPCM) calculations were performed on the optimized gas-phase geometries. Addition of solvation effects removes the encounter complex as a minimum along the reaction coordinate (Figure 9). The separated reactants go directly to the first transition state, and the activation barrier is significantly lowered. The activation barrier ranged from 0.21 to 3.16 kcal/mole. Once past the transition state, the bicarbonate is formed. When including solvation effects, the energy differences between the Lindskog (I1) and Lipscomb (I2) intermediates are much smaller. In the gas-phase the energy difference was ∼5 kcal/mole or greater, but in solution the energy differences are reduced and ranged from 0.18 to 2.31 kcal/mole. Addition of a water molecule to the intermediate structures does not significantly change the energy difference between the two geometries for the CPCM calculations. In some cases, the activation barrier for interconversion of I1 to I2 is the highest barrier.

The activation barrier for bicarbonate release is significantly reduced by solvent effects. In the case of the **N3** complexes, the activation barrier is reduced by 12.48 and 11.56 kcal/mole for **N3**-Zn and **N3**-Co, respectively. Solvation in the high dielectric medium makes separation of the two charged species more favorable, and the reduction in the barrier is large enough that in the case of **N3**-metal ion, interconversion of I1 to I2 is rate-limiting. Having the activation barrier for interconversion between the two intermediate structures as the rate-limiting step for this reaction is an unexpected result, and may be an artifact of using the gas-phase geometries. This is the only transition state (TS2) along the reaction coordinate that does not involve either formation or breaking a bond. TS1 and TS3 maybe over stabilized when using the gas-phase geometries. A similar result occurred in a computational study of CO_2_ being converted to HCO_3_^-^ by a ζ-carbonic anhydrase containing either cadmium or zinc [52]. In the gas-phase, nucleophilic attack of CO_2_ had the highest activation barrier, but when the dielectric constant was set to 4 (to better simulate the protein environment), the rotation barrier was rate-limiting. Interestingly, when the dielectric constant was set to 80, the rate-limiting step was again the nucleophilic attack. It should also be noted that we chose to model the interconversion of the intermediates by rotation around the bicarbonate for computational ease. Since the bicarbonate is solvent exposed on these mimetics, proton exchange with water might be a lower energy route for interconversion.

With the inclusion of solvation, the activation barrier for bicarbonate release is almost identical for either the Lindskog or Lipscomb intermediates for both **N3** complexes and **N4**-Zn. The active site of HCAII has a well ordered solvent network that provides a route for proton release to bulk solvent [80], but this network may also contribute in lowering the barrier for product release. The CPCM activation barriers for product release from Zn^2+^ overestimate the stabilization for the calculated barrier for bicarbonate dissociation in the macrocycles. Loferer et al., using QM/MM methods [81], calculated a barrier of 6.2 kcal/mole for bicarbonate dissociation in CAII, and from these calculations barriers of 3.19 (**N3**-Zn) and 4.68 kcal/mole (**N4**-Zn) were obtained. Interestingly, the activation barriers for product release are much higher for **Ph**-Zn (9.40 kcal/mole) and **Ben**-Zn (11.82 kcal/mole). Having a hydrophobic environment around the reacting species (methyl groups in **Ph** and phenyl groups in **Ben**) shows that nucleophilic attack is sensitive to solvent exposure, but the product release barrier is less affected by solvent. In fact, other than nucleophilic attack of CO_2_, the reaction profile for both **Ph** and **Ben** are relatively unchanged. In the gas-phase, the overall reaction for all species is endothermic, but with the addition of solvation effects all the reactions become exothermic with the exception of **N4**-Co.

For **N4**-Co, solvation effects did not significantly reduce the activation barrier for bicarbonate release and is the only species that is endothermic in solvent. It would appear that **N4**-Co does not catalyze the hydration of CO_2_ even though it had the lowest barrier for nucleophilic attack. The preference of an octahedral geometry for **N4**-Co makes release of bicarbonate improbable. This is consistent with experiments that showed a 5-coordinated Co^2+^ complex (four nitrogens and one oxygen from water) is able to form cobalt-hydroxide but does not catalyze the hydration of CO_2_ [82], [83]. We should also point out that having an additional water molecule coordinated to the cobalt could contribute to lowering the activation barrier or change the coordination of bicarbonate to unidentate, but we did not pursue these calculations since it was beyond the scope of the present study. Clearly, solvation has a significant effect on the activation barrier for product release, although the reduction in the barrier could be overestimated because we are not using optimized CPCM structures.

### Conclusions

Models that mimic the reactivity of carbonic anhydrase are of interest not only academically but to industry, which is trying to lower the amount of CO_2_ being released into the atmosphere [84]. Two of the most successful mimetics of carbonic anhydrase are the cyclic polyamines, 1,4,7,10-tetraazacyclododedacane (**N4**) and 1,5,9-triazacyclododedacane (**N3),** and when coordinated to zinc are able to catalyze the reversible hydration of CO_2_. From our calculations, the Zn^2+^ and Co^2+^ complexes of **N3** have very similar coordination geometries to human carbonic anhydrase II and comparable energetics. The **N4**-Zn complex has slightly higher turnover than the **N3**-Zn but has been criticized as a mimic for human carbonic anhydrase II because it has pentacoordinate geometry. Although the coordination differs, the calculations show that **N4**-Zn follows the same reaction as the **N3**-Zn/Co complexes. The **N4**-Co complex is able to lower the barrier for nucleophilic attack more than any of the other complexes by having an octahedral geometry around Co^2+^, but this is at the expense of being able to release bicarbonate later in the reaction.

Interestingly, the gamma-class carbonic anhydrase from *Methanosarcina thermophila* (Cam), which normally uses Fe^2+^ to catalyze the hydration of CO_2_, may have found a way around this product release problem [85]. This carbonic anhydrase can also utilize pentacoordinated Zn^2+^ or hexacoordinated Co^2+^, and Co-Cam is actually better at catalyzing the hydration reaction than Zn-Cam [86]. The crystal structure of bicarbonate bound in Zn-Cam and Co-Cam show they have different coordination positions around the metal ion (Figure S8) [87]. For Zn-Cam, the geometry of bicarbonate resembles the Lipscomb intermediate for **N3** with the carboxylate oxygens 2.48 and 3.11 Å from Zn^2+^. In Co-Cam, only one oxygen in bicarbonate is bound to Co^2+^, and two water molecules take up the other coordination sites. Interestingly, the geometry of bicarbonate around Co^2+^ most resembles the TS2 structure of **N4**-Co. It would be interesting if a catalyst based on the binding geometry in Cam could be created. If possible, its application to industry could be significant since the susceptibility of Zn-Cam and Co-Cam to anionic inhibitors differs [88]. The difference in the inhibitors is likely due to the coordination preference of the metals.

The activation barriers for **N3**-Zn and **N4**-Zn from our calculations are quite low yet these complexes are ∼1000 slower at catalyzing the hydration of CO_2_ relative to HCAII. One aspect of the reaction that could not be readily studied is the importance of reactant positioning. Although the rate-limiting step in HCAII is proton loss from the metal bound water, it would not be expected to be limiting for these mimetics that are solvent exposed and function optimally at alkaline pH. Recent crystal structures show that HCAII contains a hydrophobic pocket that binds CO_2_ in a conformation that will readily react with the zinc-hydroxide [54]. In fact, the presence of a metal is not even required for CO_2_ binding in HCAII. Additionally, placement of zinc coordinated by three histidines within the hydrophobic interior of an α-helical triple coiled coil showed CO_2_ hydration activity within 500-fold of HCAII [89]. Reactant positioning likely is an important aspect of the hydration reaction by HCAII and for these mimetics.

## Supporting Information

Figure S1 Relative energies for stationary points of **N4**-Zn (gray) and **N4**-Co along the reaction coordinate calculated at the B3LYP/6-311+G(d) and MPWLYP1M/6-311+G(d) (parenthesis) level of theory.
(TIF)

Figure S2 Calculated structures for the transition state (TS2) separating the Lindskog (I1) and Lipscomb (I2) intermediates for **N4**-Zn (A) and **N4**-Co (B). The angle listed is formed by the point generated by the center of mass of the ring nitrogens-metal ion-coordinating oxygen of bicarbonate.
(TIF)

Figure S3 Optimized structures of I2 (Lipscomb intermediate) interacting with a single water molecule for **N3** (A), **N4** (B), **Ph** (C), and **Ben** (D), respectively. Numerical values are in angstroms.
(TIF)

Figure S4 The interconversion of the **Ph**-Zn complex from I3 to I4 shown from the top view and side view.
(TIF)

Figure S5 Comparison of calculated structures for **Ph**-Zn with bicarbonate and X-ray crystal structures of carbonic anhydrase II interacting with acetate. Panels (A) and (B) show wild-type carbonic anhydrase II and Panel (C) show the E106Q mutant.
(TIF)

Figure S6 Overlay of the wild-type human carbonic anhydrase coordinated to acetate (1CAY, green) and the mutant T199A of human carbonic anhydrase coordinated to bicarbonate (1CAM, cyan).
(TIF)

Figure S7 Measured initial rate of Zn-STBI (sulfonated-**Ben**) and Zn-Cyclen (**N4**) in TAPS buffer at 25° and 50°C for CO_2_ hydrolysis reaction.
(TIF)

Figure S8 X-ray crystal structures of the active site of Zn-Cam (1QRL) and Co-Cam (1QRE) binding bicarbonate, are shown in (A) and (B), respectively. Numerical values are the oxygen to metal distances and are in angstroms.
(TIF)

Table S1 Imaginary frequencies calculated for the gas-phase transition states of the individual catalysts. Values are in cm-1. The TS3*-axial structure was the transition state for axial release of bicarbonate from **Ben**.
(DOCX)

Table S2 Natural population analysis for the metal ion and coordinating oxygen within the calculated catalyst. The OH species is the isolated hydroxylated catalyst. Values with asterisk are for the coordinated water oxygen. All values are in |eu|.
(DOCX)

Text S1 Additional details on the experimental characterization and kinetics measurements of the zinc complex of tris(6-sulfobenzimidazolylmethyl)amine.
(DOCX)
