# Supplementary figures and images for: Comparison and Analysis of Zinc and Cobalt-Based Systems as Catalytic Entities for the Hydration of Carbon Dioxide
Source: PLoS One. 2013 Jun 20;8(6):e66187. doi: 10.1371/journal.pone.0066187 (PMC3688778; doi:10.1371/journal.pone.0066187)

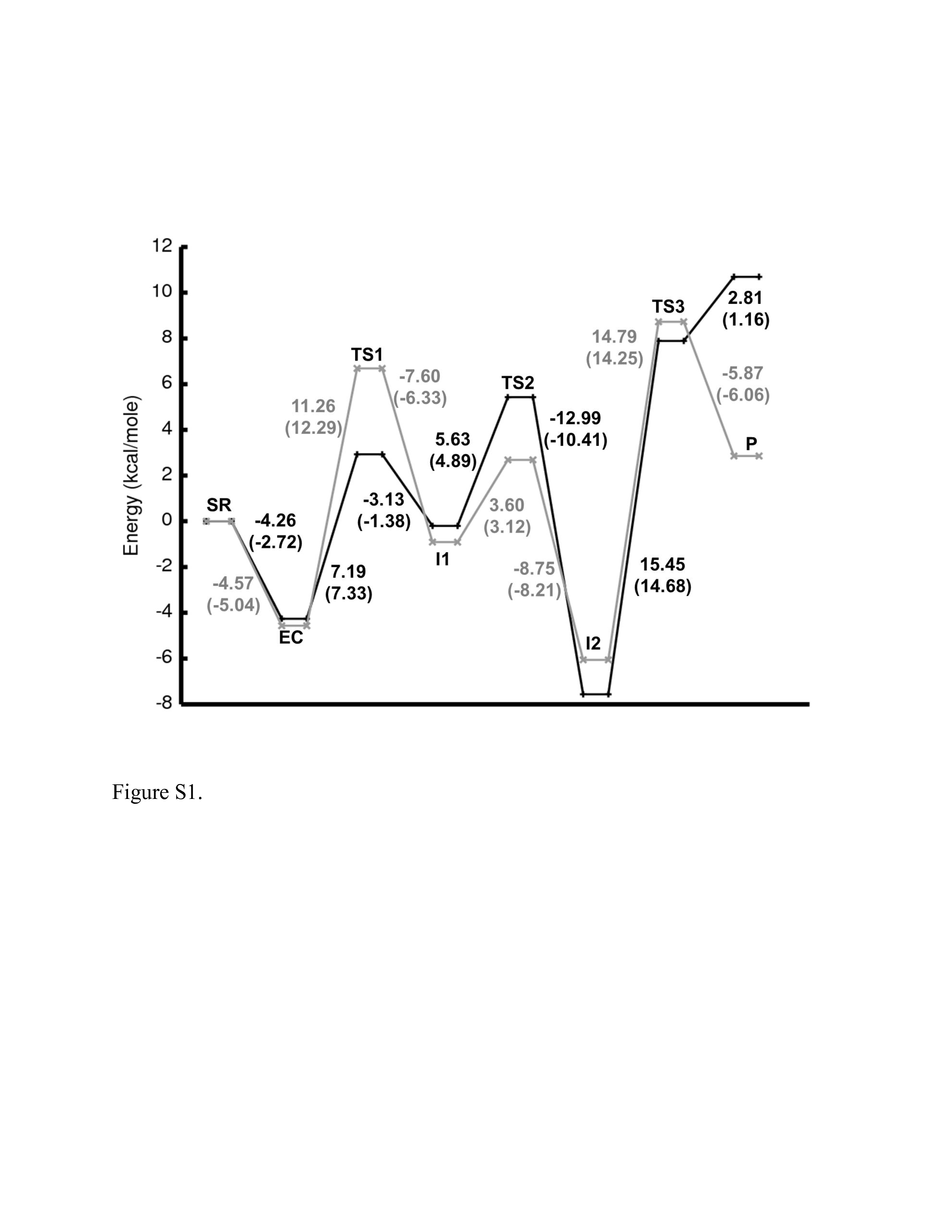

Supplement: Figure S1 — Relative energies for stationary points of N4-Zn (gray) and N4-Co along the reaction coordinate calculated at the B3LYP/6-311+G(d) and MPWLYP1M/6-311+G(d) (parenthesis) level of theory. (TIF) [file pone.0066187.s001.tif]

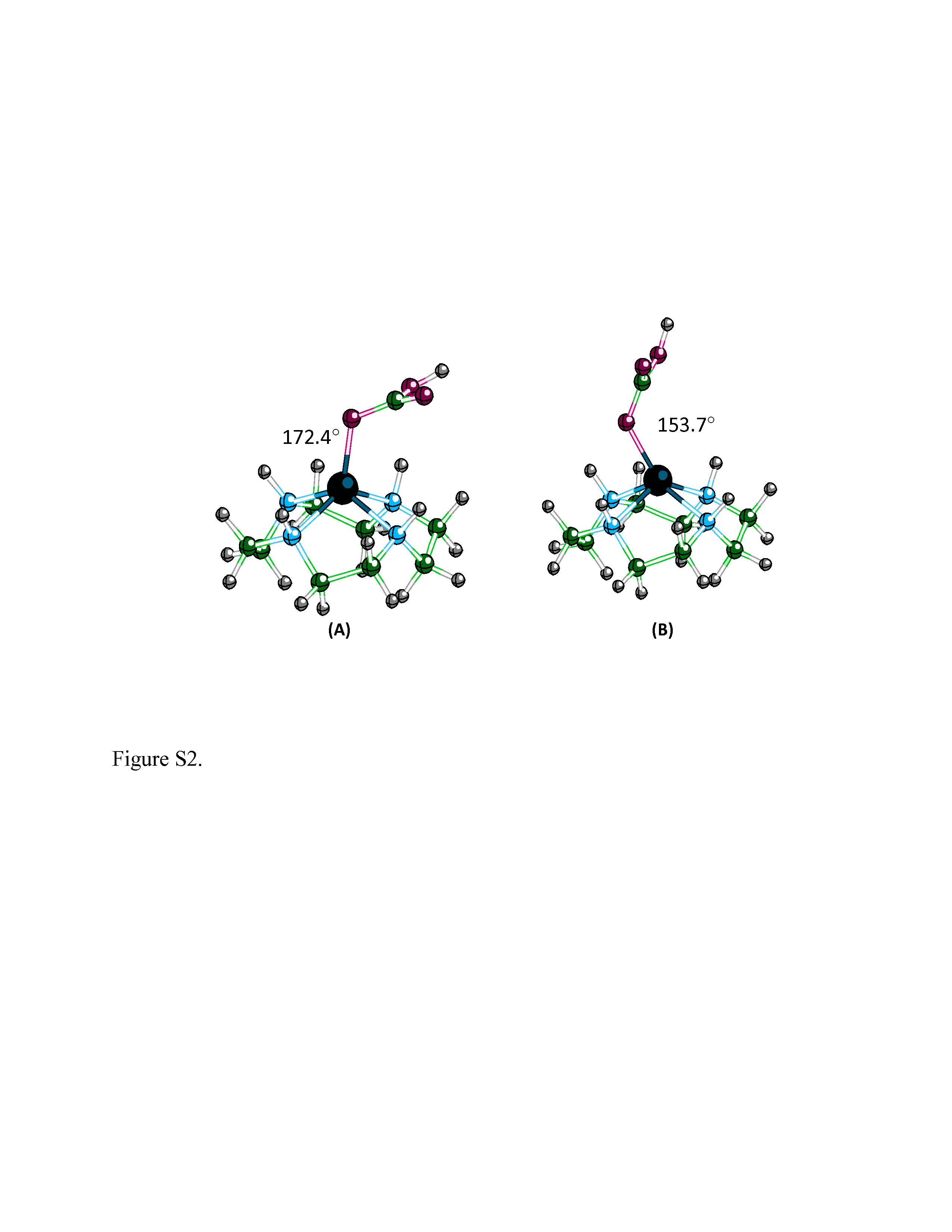

Supplement: Figure S2 — Calculated structures for the transition state (TS2) separating the Lindskog (I1) and Lipscomb (I2) intermediates for N4-Zn (A) and N4-Co (B). The angle listed is formed by the point generated by the center of mass of the ring nitrogens-metal ion-coordinating oxygen of bicarbonate. (TIF) [file pone.0066187.s002.tif]

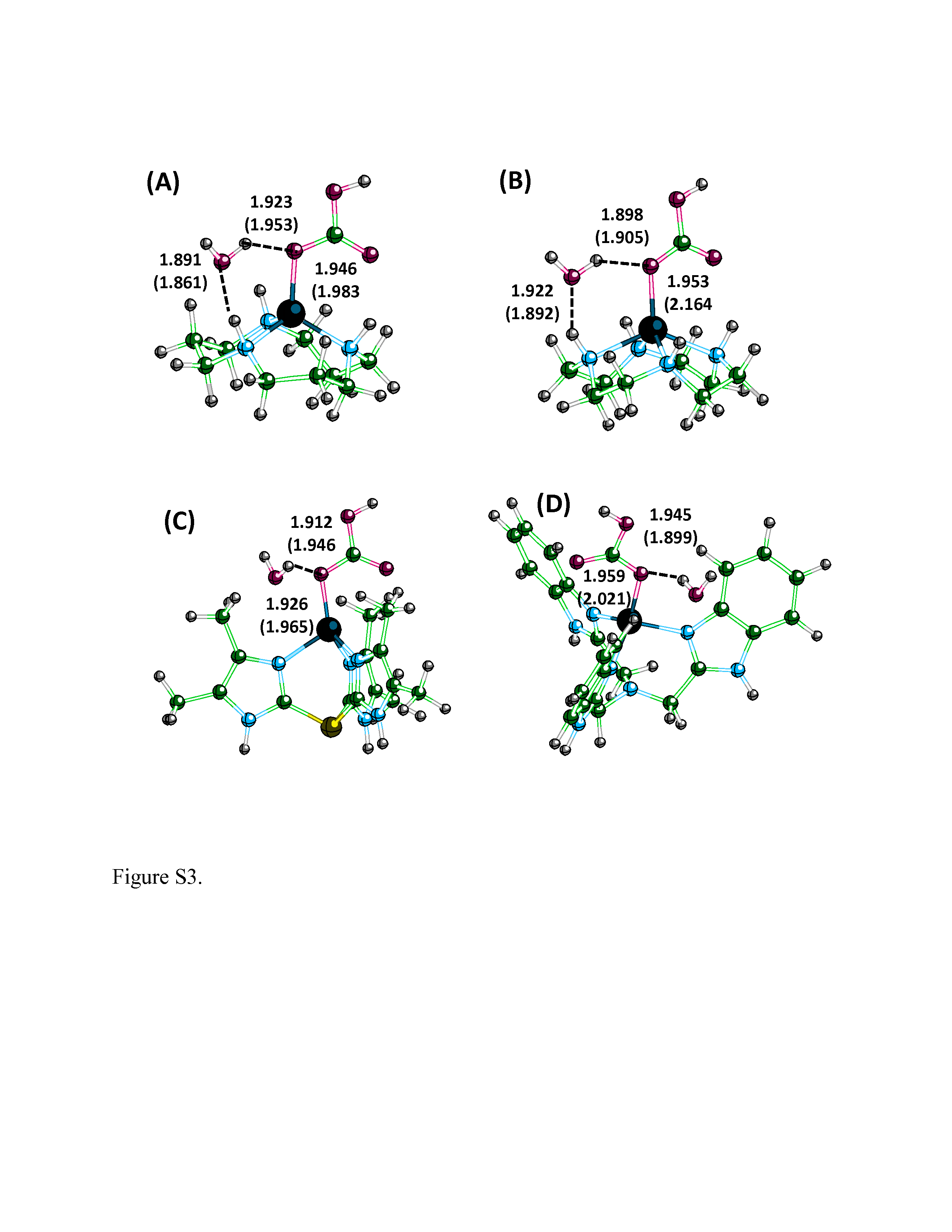

Supplement: Figure S3 — Optimized structures of I2 (Lipscomb intermediate) interacting with a single water molecule for N3 (A), N4 (B), Ph (C), and Ben (D), respectively. Numerical values are in angstroms. (TIF) [file pone.0066187.s003.tif]

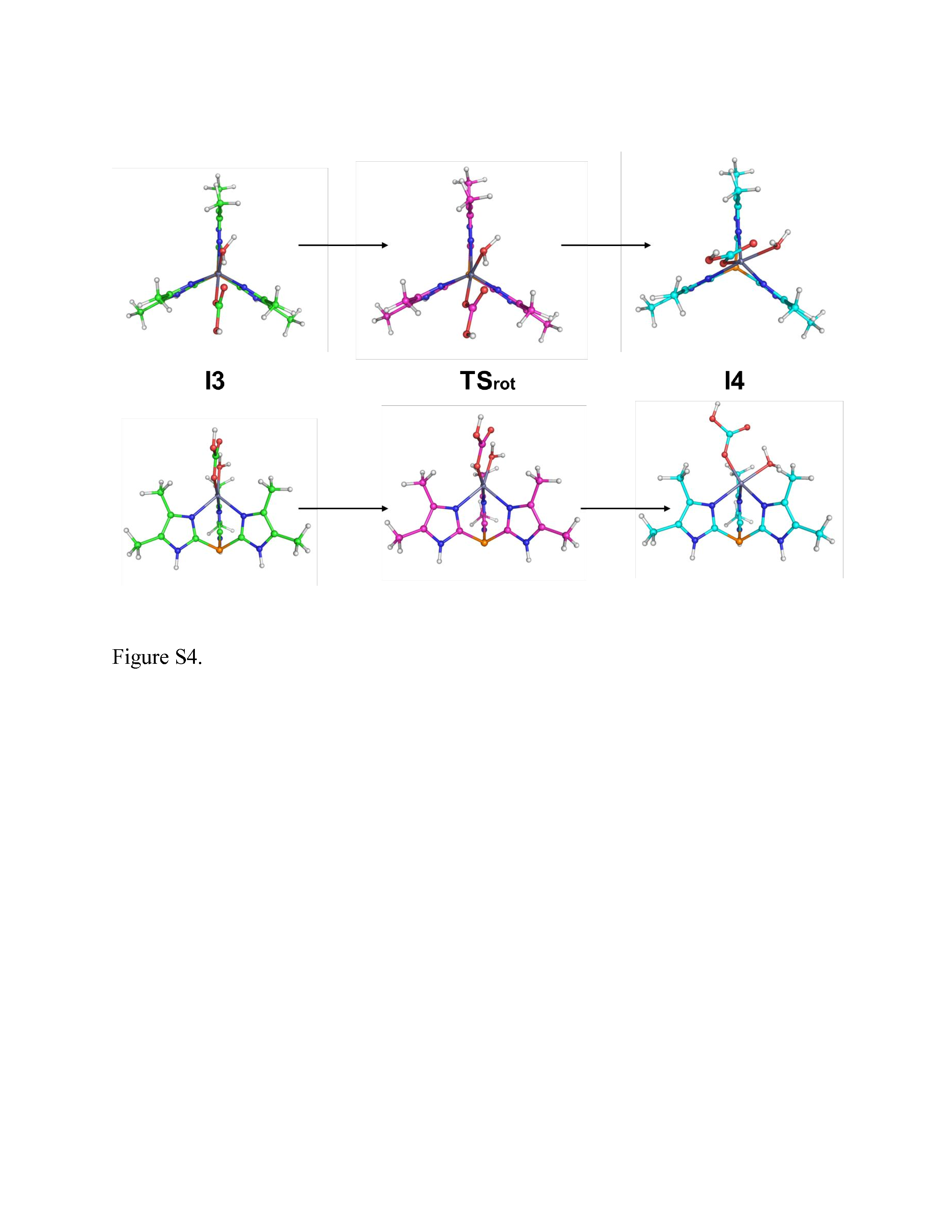

Supplement: Figure S4 — The interconversion of the Ph-Zn complex from I3 to I4 shown from the top view and side view. (TIF) [file pone.0066187.s004.tif]

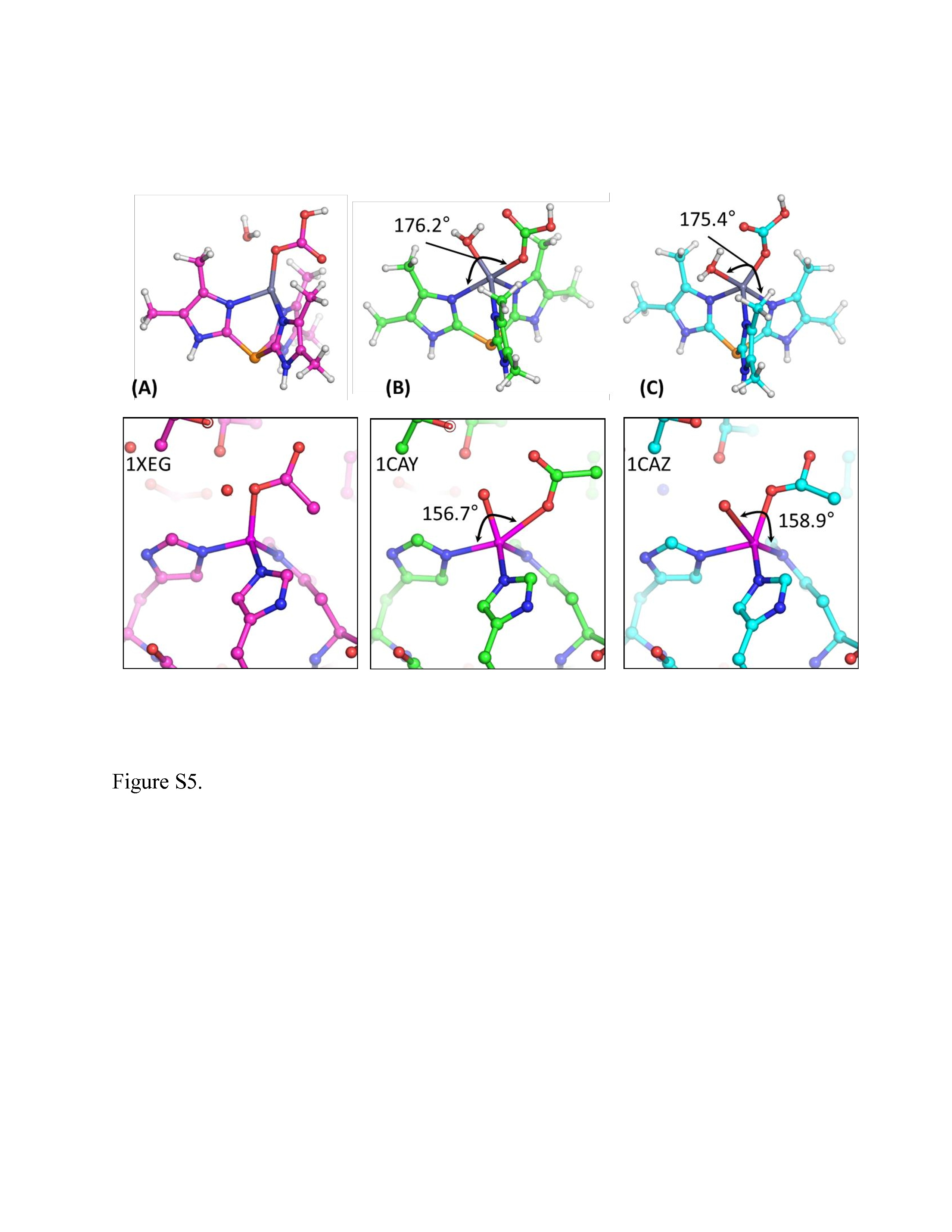

Supplement: Figure S5 — Comparison of calculated structures for Ph-Zn with bicarbonate and X-ray crystal structures of carbonic anhydrase II interacting with acetate. Panels (A) and (B) show wild-type carbonic anhydrase II and Panel (C) show the E106Q mutant. (TIF) [file pone.0066187.s005.tif]

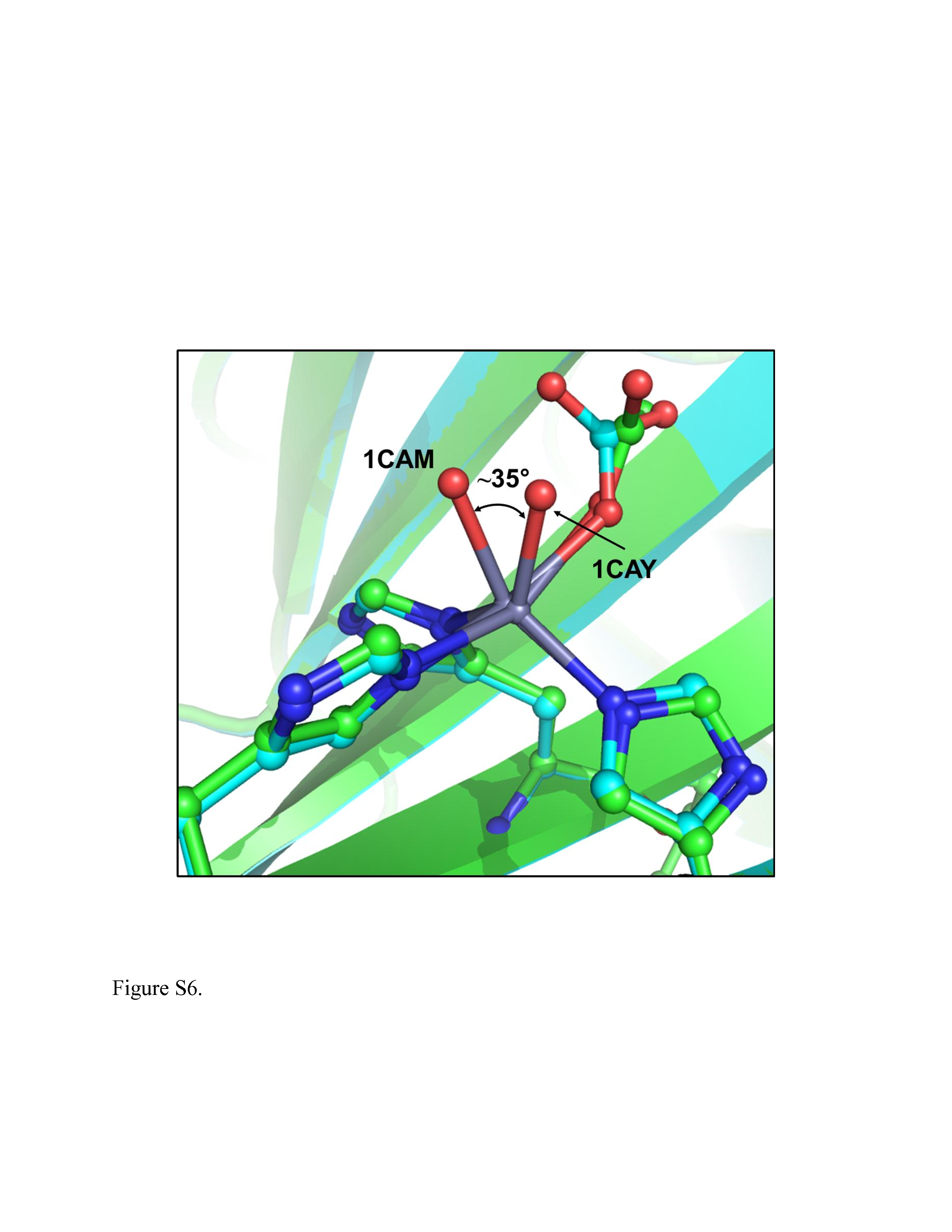

Supplement: Figure S6 — Overlay of the wild-type human carbonic anhydrase coordinated to acetate (1CAY, green) and the mutant T199A of human carbonic anhydrase coordinated to bicarbonate (1CAM, cyan). (TIF) [file pone.0066187.s006.tif]

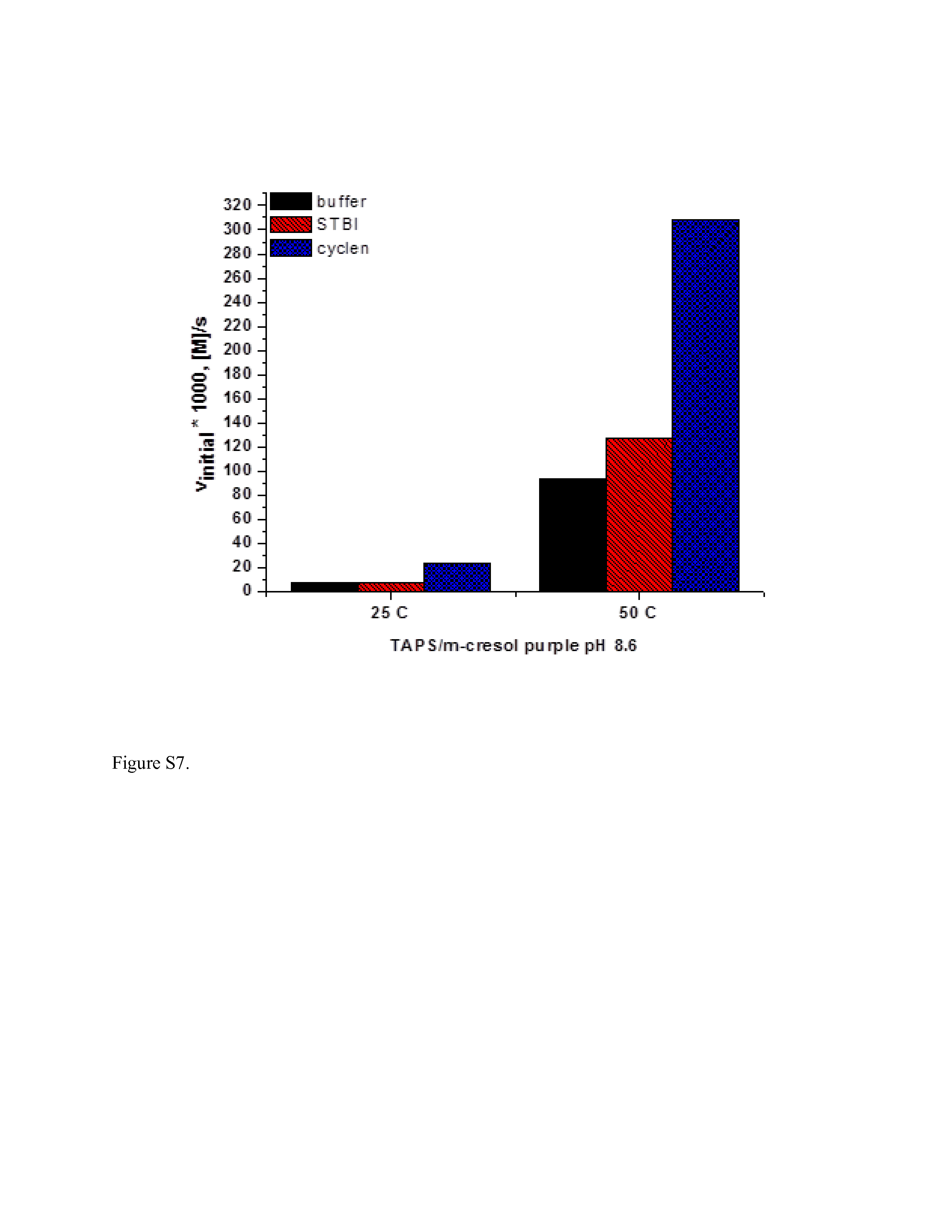

Supplement: Figure S7 — Measured initial rate of Zn-STBI (sulfonated-Ben) and Zn-Cyclen (N4) in TAPS buffer at 25° and 50°C for CO2 hydrolysis reaction. (TIF) [file pone.0066187.s007.tif]

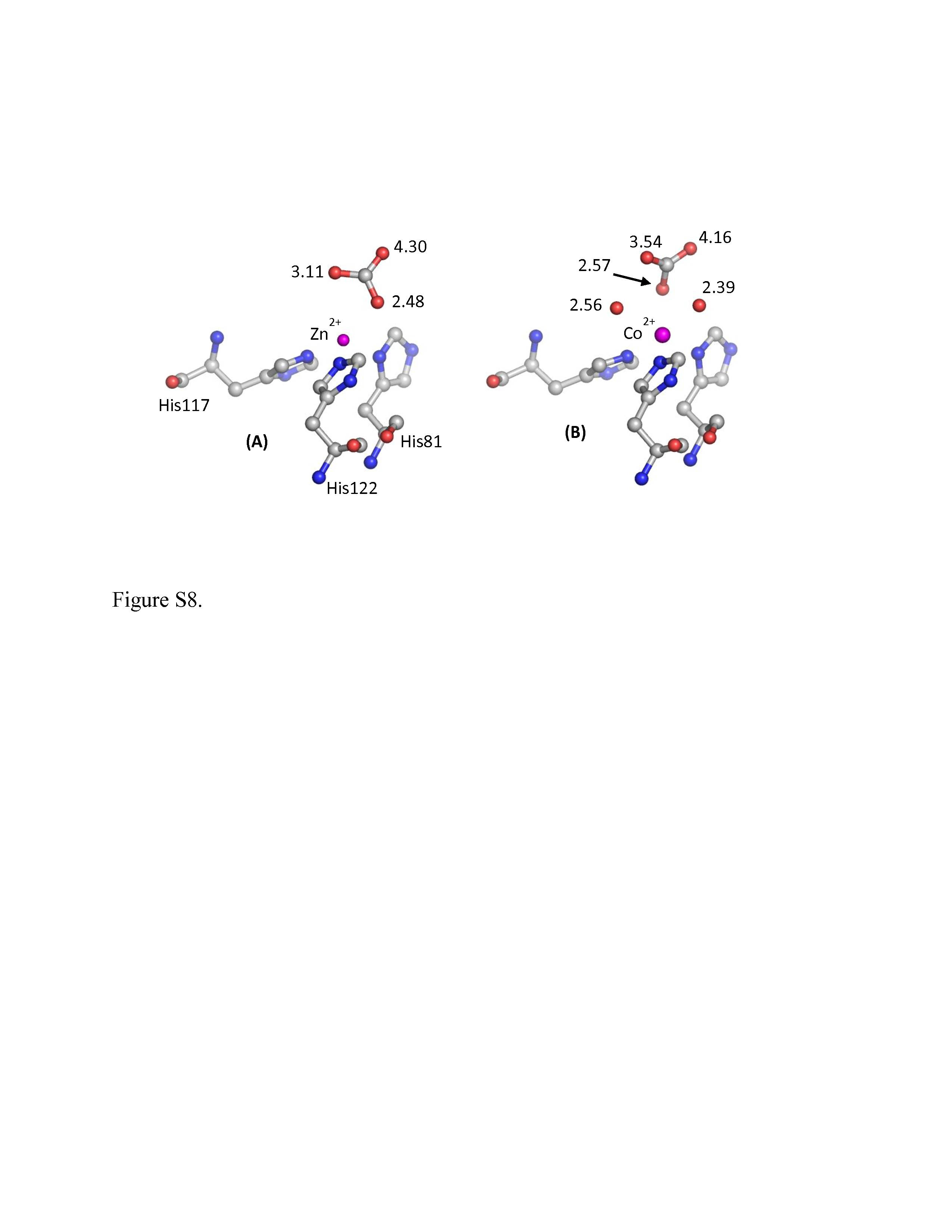

Supplement: Figure S8 — X-ray crystal structures of the active site of Zn-Cam (1QRL) and Co-Cam (1QRE) binding bicarbonate, are shown in (A) and (B), respectively. Numerical values are the oxygen to metal distances and are in angstroms. (TIF) [file pone.0066187.s008.tif]
