# Supplementary material for: Comparison and Analysis of Zinc and Cobalt-Based Systems as Catalytic Entities for the Hydration of Carbon Dioxide
Source: PLoS One. 2013 Jun 20;8(6):e66187. doi: 10.1371/journal.pone.0066187 (PMC3688778; doi:10.1371/journal.pone.0066187)
Supplement: Table S1 — Imaginary frequencies calculated for the gas-phase transition states of the individual catalysts. Values are in cm-1. The TS3*-axial structure was the transition state for axial release of bicarbonate from Ben. (DOCX) [file pone.0066187.s009.docx]

Table S1. Imaginary frequencies calculated for the gas-phase transition states of the individual catalysts. Values are in cm^-1^. The TS3*-axial structure was the transition state for axial release of bicarbonate from **Ben**.

|  | Zn^2+^ | Co^2+^ |
| --- | --- | --- |
| **N3** |  |  |
| TS1 | -186.8 | -214.8 |
| TS2 | -81.3 | -73.8 |
| TS3 | -66.7 | -108.8 |
|  |  |  |
| **N4** |  |  |
| TS1 | -140.9 | -241.6 |
| TS2 | -65.3 | -64.3 |
| TS3 | -59.9 | -758.4 |
|  |  |  |
| **Ph** |  |  |
| TS1 | -191.8 | -213.4 |
| TS2 | -61.2 | -49.9 |
| TS_turn_ | -33.0 |  |
| TS3 | -124.1 | -406.1 |
|  |  |  |
| **Ben** |  |  |
| TS1 | -155.6 | -189.6 |
| TS2 | -47.0 | -48.1 |
| TS3 | -99.2 | -110.1 |
| TS3*-axial | -75.1 | -181.1 |
