# Supplementary material for: Comparison and Analysis of Zinc and Cobalt-Based Systems as Catalytic Entities for the Hydration of Carbon Dioxide
Source: PLoS One. 2013 Jun 20;8(6):e66187. doi: 10.1371/journal.pone.0066187 (PMC3688778; doi:10.1371/journal.pone.0066187)
Supplement: Table S2 — Natural population analysis for the metal ion and coordinating oxygen within the calculated catalyst. The OH species is the isolated hydroxylated catalyst. Values with asterisk are for the coordinated water oxygen. All values are in |eu|. (DOCX) [file pone.0066187.s010.docx]

Table S2. Natural population analysis for the metal ion and coordinating oxygen within the calculated catalyst. The OH species is the isolated hydroxylated catalyst. Values with asterix are for the coordinated water oxygen. All values are in |eu|.

Zn^2+^ OC Co^2+^ OC

**N3**

OH 1.601 -1.301 1.334 -1.163

EC 1.615 -1.305 1.348 -1.179

TS1 1.638 -1.092 1.404 -1.049

I1 1.615 -0.948 1.372 -0.873

I2 1.608 -0.921 1.370 -0.803

TS3 1.615 -0.811 1.393 -0.814

-1.063* -1.034*

**N4**

OH 1.602 -1.300 1.333 -1.178

EC 1.607 -1.308 1.356 -1.192

TS1 1.621 -1.047 1.386 -1.098

I1 1.608 -0.938 1.368 -0.848

I2 1.601 -0.910 1.357 -0.781

TS3 1.602 -0.859 1.386 -0.786

-1.040* -1.153*

**Ph**

OH 1.643 -1.281 1.405 -1.142

EC 1.647 -1.286 1.417 -1.156

TS1 1.663 -1.086 1.463 -1.036

I1 1.650 -0.934 1.438 -0.856

I2 1.641 -0.894 1.434 -0.803

I3 1.660 -0.905 1.469 -0.851

-1.014* -0.990*

TS3 1.655 -0.861 1.462 -0.836

-1.067* -1.038*

**Ben**

OH 1.649 -1.298 1.405 -1.171

EC 1.651 -1.305 1.411 -1.186

TS1 1.652 -1.046 1.433 -1.016

I1 1.649 -0.946 1.433 -0.881

I2 1.645 -0.932 1.436 -0.871

I3 1.644 -0.905 1.4316 -0.859

-0.996* -0.968*

TS3 1.639 -0.874 1.144 -0.829

-1.034* -0.965*
