# Supplementary material for: Comparison and Analysis of Zinc and Cobalt-Based Systems as Catalytic Entities for the Hydration of Carbon Dioxide
Source: PLoS One. 2013 Jun 20;8(6):e66187. doi: 10.1371/journal.pone.0066187 (PMC3688778; doi:10.1371/journal.pone.0066187)
Supplement: Text S1 — Additional details on the experimental characterization and kinetics measurements of the zinc complex of tris(6-sulfobenzimidazolylmethyl)amine. (DOCX) [file pone.0066187.s011.docx]

**Supporting information for**

**Comparison and analysis of zinc and cobalt-based systems as catalytic entities for the hydration of carbon dioxide.**

Edmond Y. Lau, Sergio E. Wong, Sarah E. Baker, Jane P. Bearinger, Lukasz Koziol,

Carlos A. Valdez, Joseph H. Satcher Jr, Roger D. Aines* and Felice C. Lightstone*

Physical and Life Sciences Directorate, Lawrence Livermore National Laboratory,

Livermore, California, United States of America

Corresponding authors [aines1@llnl.gov](mailto:aines1@llnl.gov) and felice@llnl.gov

**Text S1**

**Materials.** All reagents used were of analytical grade, were purchased from commercial suppliers and were used as received. *o*-Phenylenediamine was purchased from Alfa Aesar (Ward Hill, MA). ^1^H NMR (600 MHz) and ^13^C NMR (150 MHz) spectra were recorded in D_2_O unless otherwise specified with a Bruker Avance AVB-600 nuclear magnetic resonance (NMR) spectrometer equipped with a 5 mm *z*-gradient broadband probe. Data are presented as follows: chemical shift (parts per million, ppm), multiplicity s = singlet, d = doublet, m = multiplet, br = broad, coupling constant, *J* (in hertz). Elemental analyses were obtained at the LLNL Microanalytical Facility.

**Zinc complex of Tris(6-sulfobenzimidazolylmethyl)amine [**sulfonated**-Ben]**. The sodium salt of tris(6-sulfobenzimidazolylmethyl)amine (300 mg, 0.42 mmol) was dissolved in deionized water (5 mL) in a 25 mL round bottom flask. The light grey solution was warmed to 50 ^o^C and treated with zinc(II) perchlorate hexahydrate (208 mg, 0.56 mmol) in deionized water (1 mL). The resulting solution was stirred at 50 ^o^C for 2 h and then at ambient temperature for 1 h. The flask was evaporated and the light grey solid was dried under vacuum at ambient temperature overnight to obtain the zinc(II) containing complex (397 mg, 95%). ^1^H NMR data matched the one previously published [1] while the ^13^C NMR contained multiple peaks due to the presence of all the possible isomeric complexes. Anal. (C_24_H_22_Cl_2_N_7_Na_3_O_19_S_3_Zn•2H_2_O) C, 28.43; H, 2.19; N, 6.99; Found: C, 28.92; H, 2.42; N, 7.02.

**Stopped-Flow Measurements**

Experimental catalytic rate constants for the CO_2_ hydration reaction catalyzed by tris(6-sulfobenzimidazolylmethyl)amine-Zn(II) were determined using stopped-flow spectrophotometry using methods similar to those previously described [2].

1. Nakata, K.; Shimomura, N.; Shiina, N.; Izumi, M.; Ichikawa, K.; Shiro, M. “Kinetic study of catalytic CO_2_ hydration by water-soluble model compound of carbonic anhydrase and anion inhibition effect on CO_2_ hydration.” *J. Inorg. Biochem.* **2002***, 89*, 255-266.

2. Koziol, L., Valdez, C. A., Baker, S.E., Lau, E. Y., Floyd, III, W. C., Wong, S. E., Satcher, Jr., J. H., Lightstone, F. C., Aines, R. D. “Toward a small molecule, biomimetic carbonic anhydrase model: theoretical and experimental investigations of a panel of zinc(II) aza-macrocyclic catalysts.” *Inorg. Chem*., **2012**, *51*, 6803-6812.
